# Abnormally elevated USP37 expression in breast cancer stem cells regulates stemness, epithelial-mesenchymal transition and cisplatin sensitivity

**DOI:** 10.1186/s13046-018-0934-9

**Published:** 2018-11-27

**Authors:** Tao Qin, Bai Li, Xiaoyue Feng, Shujun Fan, Lei Liu, Dandan Liu, Jun Mao, Ying Lu, Jinfeng Yang, Xiaotang Yu, Qingqing Zhang, Jun Zhang, Bo Song, Man Li, Lianhong Li

**Affiliations:** 1 Department of Pathology, Dalian Medical University, Dalian, 116044 People’s Republic of China; 2 The Key Laboratory of Tumor Stem Cell Research of Liaoning Province, Dalian Medical University, Dalian, 116044 People’s Republic of China; 3 Department of Urology, The First Affiliated Hospital of Dalian Medical University, Dalian, People’s Republic of China; 4 Teaching Laboratory of Morphology, Dalian Medical University, Dalian, 116044 People’s Republic of China; 5 Department of Pathology, Xiangyang Central Hospital, Xiangyang, 441000 People’s Republic of China; 6 Department of Dean, Dalian Medical University, Dalian, 116044 People’s Republic of China; 7 Department of Oncology, The Second Affiliated Hospital of Dalian Medical University, Dalian, 116023 Liaoning Province People’s Republic of China

**Keywords:** USP37, Stemness, EMT, Cisplatin, Hedgehog, Breast cancer

## Abstract

**Background:**

Recent studies have indicated that deubiquitinating enzymes (DUBs) are related to the stem-cell pathway network and chemo-resistance in cancer. Ubiquitin-specific peptidase 37 (USP37), a novel DUB, was identified to be a potential factor associated with tumor progression. However, the biological functions of USP37 in breast cancer remain unclear.

**Methods:**

The distribution of USP37 expression in breast cancer and the correlation between USP37 expression and the overall survival rate were detected by The Cancer Genome Atlas (TCGA) database. Gene set enrichment analysis (GSEA) was utilized to evaluate potential mechanism of USP37 in breast cancer. The USP37 expression in breast cancer tissues and breast cancer cell lines were detected by immunohistochemistry and western blotting. Sorting of breast cancer stem cells (BCSCs) were by using MACS assay. In vitro and in vivo assays were performed to examine the biological functions of USP37 in breast cancer cells. MG132, CHX chase, immunofluorescence staining and co-immunoprecipitation assays were used to test the interaction between USP37 and Gli-1.

**Results:**

Bioinformatics analysis demonstrated that USP37 gene was elevated in breast cancer tissues and its overexpression was strongly correlated with the increased mortality rate. GSEA analysis showed that USP37 expression was positively associated with cell growth and metastasis while negatively related to cell apoptosis in the TCGA breast cancer samples. USP37 expression was elevated in breast cancer tissues and breast cancer cell lines. Moreover, we also detected that USP37 was overexpressed in BCSCs. USP37 regulated the ability of cell invasion, epithelial-mesenchymal transition (EMT), stemness and cisplatin sensitivity in breast cancer cell lines. Additionally, USP37 knockdown inhibited tumorigenicity and increased anticancer effect of cisplatin in vivo. Knockdown of USP37 significantly decreased hedgehog (Hh) pathway components Smo and Gli-1. Gli-1 was stabilized by USP37 and they interacted with each other. Further studies indicated that USP37 knockdown could inhibit the stemness, cell invasion and EMT in breast cancer via downregulation of Hh pathway.

**Conclusions:**

These findings reveal that USP37 is highly expressed in BCSCs and is correlated with poor prognosis in breast cancer patients. USP37 can regulate the stemness, cell invasion and EMT via Hh pathway, and decreased USP37 confers sensitivity to cisplatin in breast cancer cells. USP37 is required for the regulation of breast cancer progression, as well as a critical target for clinical treatment of breast cancer.

**Electronic supplementary material:**

The online version of this article (10.1186/s13046-018-0934-9) contains supplementary material, which is available to authorized users.

## Introduction

Breast cancer is the deadliest form of carcinoma affecting women, with nearly a quarter-million cases diagnosed in 2016 [1]. Although there are effective treatments against some types of breast carcinoma, such as those for subtypes with abnormal overexpression of the HER2/Neu oncogene, the majority breast cancers remain incurable. Breast cancer stem cells (BCSCs) possess self-renewal and differentiation capabilities, leading to tumor recurrence, metastasis and therapeutic resistance [2, 3]. CD44^+^/CD24^−^ or aldehyde dehydrogenase1 (ALDH1) phenotypes are efficient in the identification of BCSCs from breast cancer populations. However, there is a small overlap between CD44^+^/CD24^−^ and ALDH1 stem phenotypes, as well as less stem markers in differentiation of different breast cancer subtypes [4]. Therefore, it is necessary to identify more discriminatory biomarkers of distinct molecular subtypes for the isolation and identification of the BCSCs subpopulation.

EMT describes the process by which epithelial cells detach from neighboring cells and are transferred to other tissue sites via dissolution of basement membrane and passage through the extra-cellular matrix [5, 6]. EMT could also facilitate the generation of cancer stem cells from more differentiated cancer cells [7]. The EMT process during breast carcinogenesis is considered to be controlled by a series of signaling pathways, including Notch, Wnt/β-catenin and Hedgehog [8, 9]. The Hedgehog (Hh) pathway is responsible for the maintenance of stem cells and EMT, which can contribute to the evolution of breast cancer [10].

Ubiquitination describes a highly conserved and reversible modification process of protein degradation, which is involved in nearly all aspects of cell biology [11]. Deubiquitinating enzymes (DUBs) can prevent ubiquitin-mediated degradation of target proteins [12]. Importantly, dysregulated DUBs expression is frequently associated with the tumorigenesis process, specifically cell self-renewal, apoptosis and EMT [13, 14]. It has been confirmed that DUBs are essential for the regulation of stem cell-related markers and controlling various steps of metastatic progression, including invasion, dissemination and eventual metastasis to distant organs [14, 15]. Ubiquitin specific peptidase 37 (USP37), a novel DUB, is a member of ubiquitin-specific processing proteases family. Human USP37, localized mainly in the cytoplasm, is composed of 979 amino acids harboring three ubiquitin-interacting motifs between the Cys box and His box of the primary sequence [16, 17]. The function of USP37 was initially identified as a potent regulator of the cell cycle where it could accelerate the G1/S transition with exceptional high expression [18, 19]. Previous studies have found that USP37 could regulate the stem cell-related marker SOX2 by binding with its promoter region at the transcriptional level [20]. Pan et al. reported that high levels of USP37 gene expression in lung cancer promoted cell viability as well as the Warburg effect via deubiquitination and stabilization of pluripotent factor c-Myc protein [21]. These advances implicated that USP37 gene may be associated with the stemness of tumor cells facilitating cancer progression. Recent studies indicate that USP37 is a potential factor involved in breast cancer progression [16]. However, the biological function of USP37 in the direct regulation of BCSCs and EMT remains unexplored.

In this study, we found that USP37 expression was upregulated in breast cancer tissues compared with surrounding tissues and its overexpression was significantly correlated with increased rates of mortality. We demonstrated that USP37 was highly expressed in BCSCs. The knockdown of USP37 could inhibit the stemness, cell invasion and EMT via downregulation of Hedgehog pathway. USP37 also interacted with and stabilized glioma-associated oncogene 1 (Gli-1) protein. Additionally, USP37 knockdown enhanced the sensitivity of breast cancer cells to cisplatin in vitro and in vivo. We postulate that USP37 may represent a novel molecular target for breast cancer treatment.

## Materials and methods

### Bioinformatic analysis

Gene data extracted from invasive breast tumor samples was obtained from the TCGA Data Portal. Results of analysis were used to create figures via GraphPad Prism. According to PAM50 gene expression signature, information about four breast cancer subtypes (basal-like, Luminal A, Luminal B, and enriched Her-2) were classified. A scatter plot diagram, where each dot indicated an individual sample, was synthesized using the All Complete Tumors of Breast Invasive Cancer dataset [22, 23]. For survival analysis, clinical data related to invasive breast carcinoma were downloaded from the TCGA database [22]. Kaplan–Meier curves were analyzed by GraphPad Prism.

Gene set enrichment analysis (GSEA) was utilized to evaluate potentially biological mechanism associated with USP37 mRNA expression levels in the TCGA breast cancer samples. GSEA software which was obtained from the Broad Institute draws the result pictures automatically.

### Cell culture and animals

Human normal breast epithelial cells (MCF-10A) and breast cancer cell lines (MCF-7, MDA-MB-231, BT549 and T47D) were obtained from the Laboratory of Pathology at Dalian, Medical University. The composite culture of MCF-10A cells used DMEM/F12 media supplemented with insulin (10 μg/mL), cholera toxin (100 ng/mL) (Sigma-Aldrich), EGF (20 ng/mL) (R&D systems, Wiesbaden, Germany), hydrocortisone (500 ng/mL), and L-glutamine (Invitrogen, Gaithersburg, MD, USA). MCF-7 cells were cultured in DMEM/HIGH GLUCOSE (Hyclone, Logan, UT, USA) with 10% FBS. MDA-MB-231 cells were cultured in MEM Alpha Modification medium (Hyclone) supplemented with 10% FBS. In addition, BT549 and T-47D cells were cultured in RPMI-1640 medium (Hyclone) supplemented with 10% FBS. Growth condition for all cells was 37 °C at 5% concentration of CO_2_.

BALB/c Nude mice (6 to 8 weeks old) were purchased from Vital River Laboratory Animal Technology Company (Beijing, China) and reared according to the Animal Care and Use Committee of Dalian Medical University.

### Plasmids, antibodies and reagents

The pEZ-M35-USP37 plasmid was created by FulenGen Co. (Guangzhou, China). Anti-Gli-1 and anti-Smoothened antibodies were obtained from Abcam (Cambridge, MA, USA); all other antibodies were obtained from Proteintech Group, Inc. (Wuhan, China). Secondary antibodies were purchased from Santa Cruz Biotechnology (Dallas, TX, USA). Cycloheximide (CHX), MG132, cisplatin and purmorphamine reagents were obtained from MedChemExpress (Shanghai, China).

### siRNA, shRNA, lentivirus

The design and synthesis of siRNAs were completed by RiboBio company (Guangzhou, China), and transfection of siRNAs against USP37 was performed with Lipofectamine 2000. MCF-7 and MDA-MB-231 cells were transfected with USP37 siRNAs or a negative control (NC) siRNA. Lentivirus vectors including short hairpin RNA against USP37 (shUSP37#2) and the negative control (shScramble) were purchased from GenePharma Company (Shanghai, China). The transfected cells were treated with puromycin (Clontech, USA) for selection. To overexpress USP37 in MCF-7 cells, the pEZ-M35-USP37 plasmid (2 μg) were infected into MCF-7 cells via Lipofectamine 2000. Expression was confirmed by RT-qPCR and western blotting. The siRNA or shRNA sequences were listed in Additional file 1: Table S1.

### BCSCs isolation

Here, 1 × 10^7^ MCF-7 cells were grown with primary antibody against CD24 following the manufacturer’s protocol for the CD24 Microbead Kit (Miltenyi Biotec, Bergisch Gladbach, Germany). Labeled cells were then incubated with goat anti-mouse IgG microBeads (Miltenyi Biotec) and magnetically separated with MiniMACS columns (Miltenyi Biotec). Acquired CD24^−^ cells were incubated with CD44 microbeads (Miltenyi Biotec) at 4 °C for 15 mins. Cells were again washed and magnetically separated.

### Colony formation assay and cell viability assay

Colony formation assay of breast cancer cells was carried out by plating infected cells at a density of 1000 cells/well in a 6-well plate and then different concentrations of cisplatin were added. After 2 weeks of incubation, cells were washed three times with phosphate buffered saline, fixed with methanol and stained with 1% crystal violet. Visible colonies were stained violet and counted for data analysis. The detection of cell viability was performed in accordance with the Cell Counting Kit 8 assay (Dojindo Laboratories, Kumamoto, Japan).

### Cell invasion assay

Cells were similarly cultured in MEM Alpha Modification medium for 24 hours. Briefly, 1 × 10^5^ cells were seeded without serum into 24-well insert Transwell chambers (8 μm pore size, Corning, USA) and pretreated with Matrigel (BD, Bioscience, San Jose, CA, USA). Medium supplemented with 20% serum was added into the lower chamber. After 12–16 hours of seeding, cotton swabs were used to clean the upper cells. The cells on the other side of the membrane were stained with 1% crystal violet. A randomly selected area was counted with an optical microscope.

### Wound healing assay

Cells were incubated in 12-well plates. When cellular density reached nearly 100%, the cell monolayer was wounded with a 200 μl micro-pipette tip. The wound areas were washed three times with phosphate buffered saline (PBS). Then the medium was changed to MEM Alpha Modification without FBS. The wound areas were micrographed at 0, 12, 24 and 48 h. All assays were performed in triplicate.

### Western blotting

Cold PBS was used to wash the transfected cells. Then cells were harvested and treated with a mixture of lysis and 1 × RIPA buffers (Sigma Chemicals, St. Louis, MO, USA). Protein concentrations were estimated using the Easy IIProtein Quantitative Kit (Transgen, China). Thirty to forty microgram of each protein lysate was separated by 8–12% sodium dodecyl sulfate-polyacrylamide gel electrophoresis (SDS-PAGE). Samples were transferred to PVDF membranes (Millipore, Billerica, MA, USA). The PVDF membranes were blocked with 5% non-fat milk dissolved in TBST (TBS and 0.01% Tween-20) for 2 h at room temperature, then incubated with the specific antibodies solution overnight at 4 °C. Finally specific antibodies were diluted as follows: USP37, 1:500; Gli-1, 1:500; Smoothened, 1:500; E-cadherin, 1:1000; Snail1, 1:500; N-cadherin, 1:1000; Vimentin, 1:500 and GAPDH (loading control), 1:1000. Finally, the transfer membranes were incubated with anti-IgG secondary antibodies (1:16000 in TBST) for 1 h at 37 °C. The protein band images were captured with a ODYSSEY infrared imaging system.

### RNA extraction and real-time quantitative PCR analysis

Total RNA was extracted from cultured cells with Trizol reagent (Transgen, China). In total, 1 μg of total RNA was reverse-transcribed with the All-in-one First-Strand cDNA Synthesis SuperMiX kit (Transgen, China). The mRNA expression levels of USP37, ALDH1, CD24 and CD44 were quantified by real-time PCR. RT-qPCR was analyzed with the iCycler™ Real Time System and a SYBR Premix EX Tag Master mixture kit (Transgen, China) according to the manufacturer’s instructions. The relative expression levels of mRNA were evaluated by using the 2^−ΔΔCt^ method. Primer sequences are listed in Additional file 1: Table S1.

### Immunofluorescence analysis

Cells were grown to 3 × 10^3^ in 24-well plates at 37 °C and fixed with 100% methanol. Next, cells were cleaned two to three times using PBST (PBS and 0.5% Triton X-100). After blocked with 3% BSA for 2 h at 4 °C, cells were incubated with the following primary antibodies (San Ying Biotechnology, China) overnight at 4 °C: USP37, 1:100; Gli-1, 1:100; Flag, 1:100; E-cadherin, 1:100 and N-cadherin, 1:100. Subsequently, cells were incubated with fluorescence conjugated secondary antibody (Sigma, 1:200) for 1 h and stained the cell nuclei with DAPI (Beyotime, China). Images were viewed with CKX41 Inverted Microscope (Olympus, Japan).

### CHX chase assay and co-immunoprecipitation (co-IP) assay

For the CHX chase assay, cells were treated with CHX (50 μg/ml) and harvested at the indicated timepoints. Treated cells were lysed, and the lysates were analyzed by western blotting with anti-USP37 or anti-GAPDH antibodies. For the Co-IP assay, 8 × 10^6^ MCF-7 cells were harvested and lysed in IP lysis buffer containing protease inhibitor. The experiment based on manufacturer’s protocols (IP Kit, Proteintech Group) was performed.

### Sphere formation assay

Cells (MCF-7, MDA-MB-231, BT549 or T47D) were inoculated into ultra-low attachment 6-well plates (Corning, New York, USA) at a density of 1000 cells/well, and then grown in DMEM/F12 supplemented with B27 (1:50, Invitrogen), 20 ng/ml human recombinant EGF (Sigma-Aldrich, St. Louis, Missouri, USA), 20 ng/ml bFGF (Sigma-Aldrich, St. Louis, Missouri, USA), 4 μg/ml heparin (Sigma-Aldrich, St. Louis, Missouri, USA), and 5 μg/ml insulin (Sigma-Aldrich, St. Louis, Missouri, USA) for 14 days. Cell colonies larger than 60 μm in diameter were counted under an inverted microscope (Olympus Corporation, Tokyo, Japan). After cultivation for 28 days, spheroid cells were collected for western blotting.

### Mouse xenograft mode

Animal experiments and procedures were conducted in accordance with the Guide for the Care and Use of Laboratory Animals (NIH). Twenty BALB/C nude mice were randomly divided into four groups. The negative control group (shScramble) and shUSP37#2-transfected MCF-7 cells (5 × 10^5^) were resuspended in 100 μl PBS and injected into mammary fat pads. When the size of tumor reached approximately 100 mm^3^, animals were randomly treated with cisplatin (2 mg/kg) or 0.9% saline. Mice were then retreated with their assigned treatment once every 2 days. The tumor sizes were measured with a Vernier caliper and recorded every other day. The tumor volume was calculated using the formula: tumor volume [mm]^3^ = (length [mm]) (width [mm])^2^ × 0.5. After the inoculation of tumors for 3 weeks, xenografted tumors were excised from sacrificed mice then analyzed by immunohistochemistry and western blotting.

### Immunohistochemical analysis

Tumor tissues were obtained from mouse xenograft mode. Paraffin-embedded tissue was cut into 5 μm thick slices that were fixed onto glass slides. All human breast cancer tissue arrays were purchased from Shanghai Outdo Biotech (Shanghai, China), including 60 cases of cancer tissues and surrounding tissues (HBre-Duc060CS-03; HBre-Duc060CS-04). These tissue sections were immunostained with corresponding antibodies, and then deparaffinized in xylene and rehydrated with ethanol. Tissue sections were preincubated with 10% normal goat serum, followed by incubation with primary antibody solution overnight at 4 °C. After washing with PBS, slides were incubated with the secondary antibody at 37 °C for 10 min, then cleaned with cold PBS and treated with peroxidase conjugated-biotin streptavidin complex for 10 min. Finally stains were examined with 3,3′-diaminobenzidine and hematoxylin. As previously described [23], the immunostained tissues were scored by multiplying the intensity (0–3) and extent (0–100) of staining.

### Ethical approval

This study was conducted with the approval of the Ethical Committee and Institutional Review Board of Dalian Medical University.

### Statistical analysis

Statistical analysis was performed with SPSS software version 11.0. Data are expressed as mean ± SD. Differences between two groups were evaluated by Student’s *t*-test. One-way ANOVA was used when comparing multiple groups. *P* < 0.05 was considered statistically significant. Clinical data analysis of survival and relevant correlations were performed with GraphPad Prism.

## Results

### USP37 is commonly overexpressed in breast cancer

**Fig. 1:**
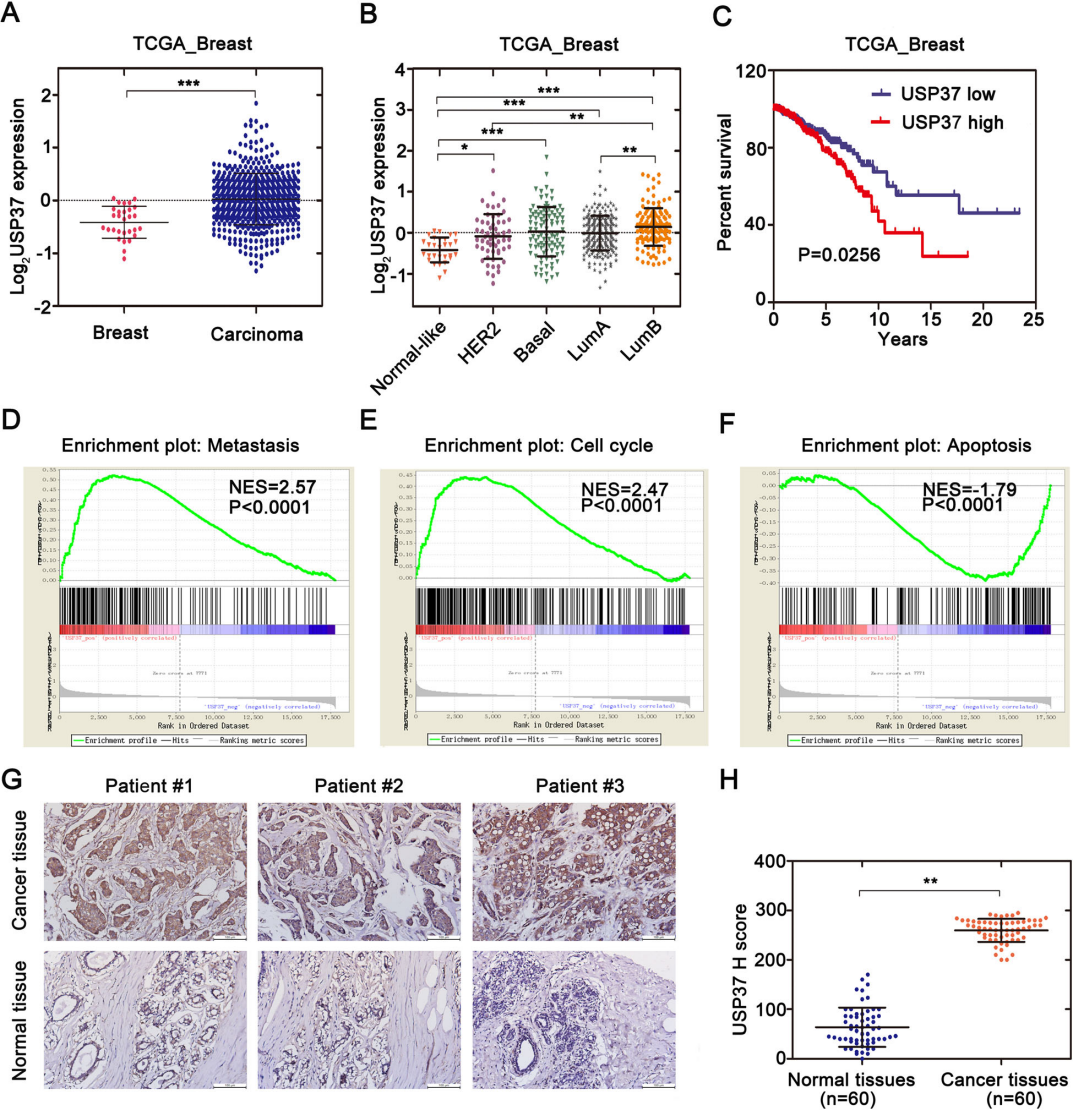
Overexpression of USP37 in human BC was correlated with poor-disease outcome. **a** Cancer with USP37 transcripts apparently had higher expression (*n* = 517) than normal breast tissues (*n* = 29) from the TCGA database [22] (***P < 0.0001). The median and interquartile range are represented by black lines. **b** USP37 transcripts among different subtypes are divided into five distinct molecular subtypes. The parameters were analyzed with the PAM50 gene expression profiling [22]. The median with interquartile range is represented by black lines. **c** Breast cancer (*n* = 738 patients) were evaluated with USP37 mRNA levels and results were correlated with overall survival over 25 years. The red line indicates patients with high USP37 transcript (*n* = 369) and blue line indicates patients with low USP37 transcript (*n* = 369). *P* value was analyzed by Kaplan-Meier analysis using GraphPad Prism. **d-f** GSEA analysis showed that USP37 expression was positively associated with metastasis (**d**) and cell growth (**e**) while negatively related to cell apoptosis (**f**) in the TCGA breast cancer samples. **g** The USP37 protein level in breast cancer tissues and surrounding tissues are shown by immunohistochemistry (IHC) (Brown: USP37). Scale bars: 100 μm. **h** USP37 IHC staining scores in breast cancer tissues (*n* = 60) and surrounding tissues (*n* = 60) are shown. **P < 0.01

**Fig. 2:**
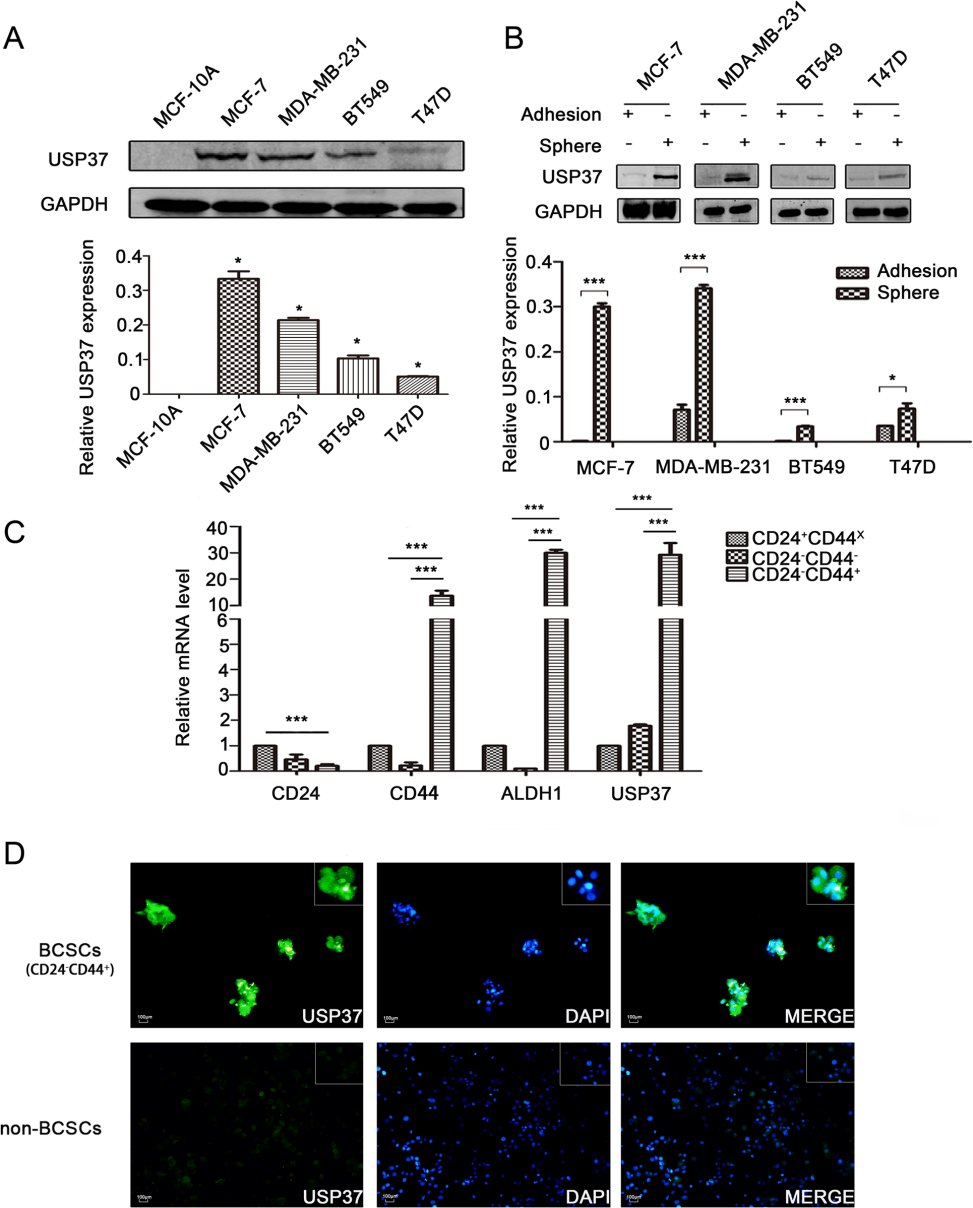
USP37 is highly expressed in breast cancer stem cells. **a** USP37 expression levels were detected in human normal breast epithelial cells (MCF-10A) and human breast cancer cells (MCF-7, MDA-MB-231, BT549 and T47D) via western blotting. *P < 0.05, **P < 0.01. **b** Protein expression levels of USP37 were tested in spheroid cells and adherent cells by western blotting. *P < 0.05, ***P < 0.001. **c** mRNA expression levels of USP37 confirmed in MCF-7 cell groups sorted by MACS by CD24 or CD44 marker by quantitative RT-PCR. ***P < 0.001. **d** Immunofluorescence staining of USP37 in BCSCs and non-BCSCs sorted by MACS with CD24 or CD44 marker in MCF-7 cells (Scale bar: 100 μm)

USP37 has been previously confirmed to be overexpressed in lung cancers cells and tissues [21]. In order to investigate the role of USP37 in tumorigenesis, we examined the breast cancer database of The Cancer Genome Atlas (TCGA) to evaluate the differential expression of USP37 [22]. Analysis of the TCGA database indicated that cancer with USP37 transcripts (*n* = 517) had a significantly higher expression level than normal samples (*n* = 29) (*P* < 0.0001) (Fig. 1a). Similar results were also found via immunohistochemistry analysis (Fig. 1g). To further confirm the overexpression of USP37 gene in breast cancer, we utilized a series of human breast cancer cells (MCF-7, MDA-MB-231, BT549 and T47D) and human normal breast epithelial cells (MCF-10A) to examine USP37 protein expression by western blotting (Fig. 2a). These results confirmed that USP37 was also overexpressed in breast cancer cells compared to normal breast epithelial cells.

For investigation of the correlation between USP37 gene and the breast cancer heterogeneity, we also tested USP37 expression in the four cell subtypes using PAM50 gene expression profiling. First, we observed an starkly different tendency within USP37 gene expression among different pathological subtypes of breast cancer cells, including the normal-like subtype having the lowest, and Luminal B type endowed with the highest expression levels of USP37 (*p* < 0.0001) (Fig. 1b). We next estimated the effect of USP37 as an oncogenic biomarker for overall survival of patients diagnosed with breast cancer. Clinical data from the TCGA database were divided into two groups according to the differential expression of USP37 gene. The results indicated that cancer with higher expression levels of USP37 was significantly correlated with the elevated rates of mortality (*P* < 0.05) (Fig. 1c). Samples with high USP37 expression also had shorter survival duration than those with low USP37 expression. Moreover, GSEA analysis showed that high USP37 expression was positively associated with metastasis, cell growth and anti-apoptosis in the TCGA breast cancer samples (Fig. 1d–f).In brief, these data confirmed that USP37 gene could act as a cancer biomarker in predicting a worse outcome for breast cancer patients. Collectively, these data suggest that USP37 is abnormally overexpressed in human breast cancer patients and cell lines.

### USP37 is highly expressed in breast cancer stem cells

CD24^−^/CD44^+^ cells and ALDH1^+^cells are widely considered to be breast cancer stem cells [24]. To validate the expression levels of the USP37 gene in breast cancer stem cells, we isolated CD24^−^/CD44^+^ cell populations from MCF-7 cell lines by magnetic activated cell sorting (MACS). We detected that USP37 was significantly overexpressed in CD24^−^/CD44^+^ cells compared to their counterpart CD24^+^ or CD24^−^CD44^−^ cells in regards to mRNA levels (Fig. 2c). Immunofluorescence staining further confirmed the upregulation of USP37 expression in BCSCs (Fig. 2d). Additionally, we used breast cancer cell lines to perform sphere-formation experiments and measured the differential expression of the USP37 gene. We confirmed that protein levels of USP37 were evidently elevated in spherical cells compared to their counterpart adherent cells (Fig. 2b). Taken together, these results indicated that elevated expression of USP37 was enriched in breast CSCs and was a novel feature of breast cancer stem cell-like subpopulation.

### USP37 knockdown suppresses breast cancer cell migration and invasion by promoting mesenchymal-epithelial transition

**Fig. 3:**
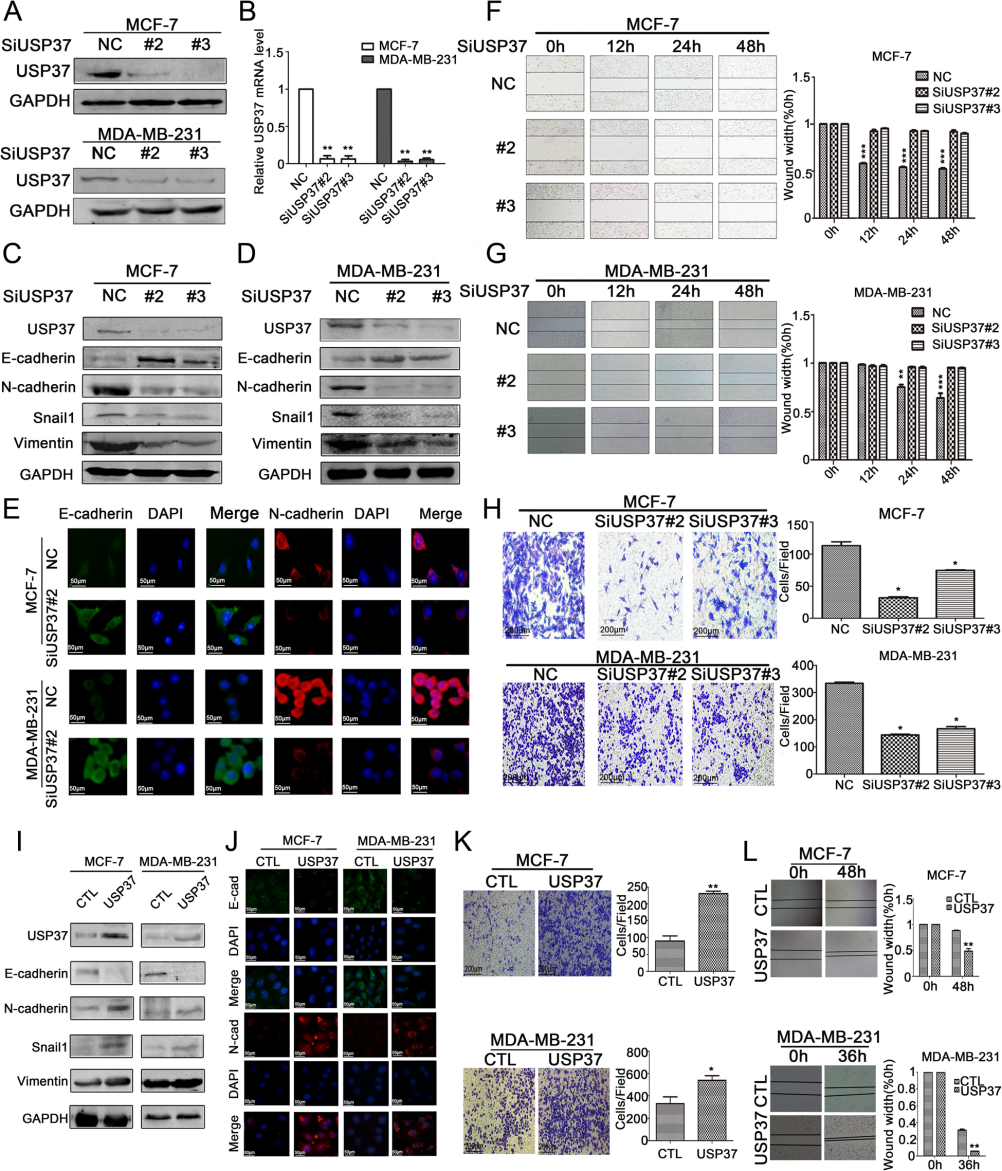
Effect of USP37 expression on breast cancer cell EMT, migration and invasion. **a** MCF-7 and MDA-MB-231 cells were transfected with USP37 siRNAs or NC siRNA for 48 h. Western blotting analysis of USP37 knockdown efficiency in MCF-7 and MDA-MB-231 cells. Relative expression levels were analyzed by Image-Pro Plus 6.0 software. **b** Real-time quantitative PCR analysis of USP37 knockdown efficiency in MCF-7 and MDA-MB-231 cells. **c**, **d** USP37 knockdown significantly decreased N-cadherin, Snail1 and Vimentin as well as increased E-cadherin expression in MCF-7 and MDA-MB-231 cells. **e** Immunofluorescence staining of E-cadherin and N-cadherin after transfection with NC siRNA or USP37 siRNA#2 (Scale bar: 50 μm). **f**, **g** Representative images of migration assays for MCF-7 and MDA-MB-231 cells after downregulation of USP37 are shown at 12, 24 and 48 h (Scale bar: 200 μm). **P < 0.01, ***P < 0.001. **h** Representative images of Matrigel-based Transwell assay for MCF-7 and MDA-MB-231 cells after USP37 knockdown are shown at the 48 h time point (Scale bar: 200 μm).*P < 0.05. **i** MCF-7 cells were transfected with control plasmid (CTL) and a USP37 overexpression plasmid (USP37) for 48 h. USP37 upregulation significantly decreased E-cadherin and increased N-cadherin, Snail1 and Vimentin expressions in both MCF-7 and MDA-MB-231 cells. **j** Immunofluorescence staining of E-cadherin and N-cadherin after USP37 upregulation (Scale bar: 50 μm). **k**, **l** Representative images of migration and Matrigel-based Transwell assay for MCF-7 and MDA-MB-231 cells after USP37 upregulation (Scale bar: 200 μm).*P < 0.05, **P < 0.01, ***P < 0.001

Currently, the underlying biological mechanism accounting for the elevated expression of USP37 in breast tumors remains unclear. To investigate the effects of USP37 on breast cancer progression, we used siRNA oligonucleotides to knockdown endogenous USP37 in MCF-7 and MDA-MB-231 cells. USP37 mRNA expression levels significantly decreased with two different siRNAs treated breast cancer cells compared to levels in the control group cells (Fig. 3b). Moreover, USP37 protein was also reduced significantly with siRNA#2 and siRNA#3 treatment of MCF-7 and MDA-MB-231 cells as evidenced by western blotting (Fig. 3a). These results confirmed that USP37 gene expression could be effectively downregulated by siRNA#2 and siRNA#3.

The expression of USP37 gene has been demonstrated to be elevated in patients with a recurrence of cancer, indicating that USP37 levels may be closely related to breast cancer distant metastasis [16]. In order to elucidate the function of USP37 in the migration and invasion of breast cancer cells, we conducted wound healing and Transwell assays to detect the role of USP37 in cell migration and invasion. Knockdown of USP37 evidently inhibited cell migration capacity in both MCF and MDA-MB-231 cells (Fig. 3f, g). The transwell assay revealed that downregulation of USP37 obviously decreased the invading capacity of MCF-7 and MDA-MB-231 cells. In addition, depletion of USP37 in MCF-7 and MDA-MB-231 cells displayed a greater reduction of invasion capacity compared to that of the control cells (Fig. 3h).

Epithelial-mesenchymal transition (EMT) is a dynamic process in which epithelial cells acquire enhanced mobility and invasive properties by losing cell-cell adhesion structures and polarity. To further detect the biological mechanism of USP37 on cell migration and invasion, we focused on the identification whether USP37 affected EMT. We examined typical EMT markers (E-cadherin, N-cadherin, Snail1 and Vimentin) by western blotting. As shown in Fig. 3c and d, knockdown of USP37 significantly decreased Snail1, N-cadherin and Vimentin expression, but increased E-cadherin expression levels. These results indicated that downregulation of USP37 induced the mesenchymal–epithelial transition (MET) process in breast cancer cell lines. Similar results were also found via immunofluorescence analysis (Fig. 3e).

Conversely, USP37 overexpression in MCF-7 cells promoted a mesenchymal phenotype with the downregulation of E-cadherin and upregulation of N-cadherin, Vimentin and Snail1 as evidenced by western blotting (Fig. 3i). Immunofluorescence assay showed that overexpression of USP37 further inhibited E-cadherin expression and upregulated N-cadherin expression (Fig. 3j). Furthermore, upregulation of USP37 markedly promoted invasion and migration of breast cancer cells (Fig. 3k and l). Based on this experimental data, we were able to further demonstrate that USP37 is involved in regulating EMT in breast cancer progression and promotes the migration and invasion capacity of breast cancer cells.

### USP37 is essential for the maintenance of cell self-renewal and chemo-resistance

**Fig. 4:**
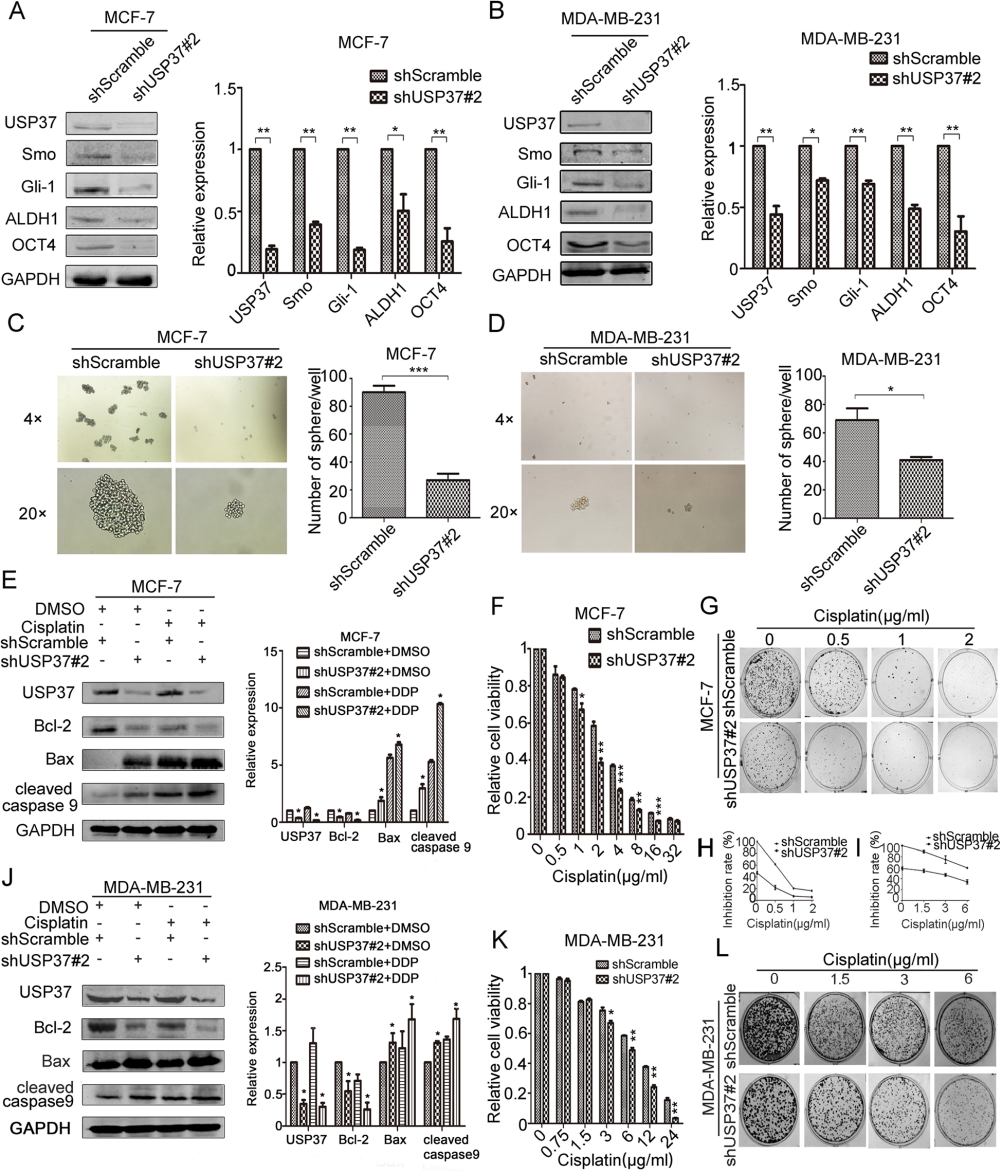
Downregulation of USP37 weakens cell self-renewal and chemo-resistance to cisplatin. **a**, **b** MCF-7 and MDA-MB-231 cells were transfected with shUSP37#2 and shScramble, and expressions of USP37, Smo, Gli-1, ALDH1 and OCT4 were confirmed by western blotting. *P < 0.05, **P < 0.01. **c**, **d** Comparison of mammosphere formation among inoculated cells in MCF-7 and MDA-MB-231 cells (original magnification, 4× or 20×).*P < 0.05, ***P < 0.001. **e** MCF-7-ShScramble and MCF-7-shUSP37#2 cells were treated with cisplatin (1 μg/ml) for 48 h, then treated cells were harvested to detect the levels of Bcl-2, Bax and cleaved caspase 9. GAPDH was examined as a loading control. **j** MDA-MB-231 cells transfected with shScramble or shUSP37#2 were exposed to 3 μg/ml cisplatin for 48 h, and the levels of Bcl-2, Bax and cleaved caspase 9 were detected via western blotting. **f**, **g**, **k**, **l** CCK-8 assay and colony formation assay showed that cell viability was decreased in MCF-7 and MDA-MB-231 cells after USP37 knockdown. Quantitation of colony formation in (**h**) MCF-7 and (**i**) MDA-MB-231. GAPDH was examined as a loading control. *P < 0.05, **P < 0.01, ***P < 0.001

**Fig. 5:**
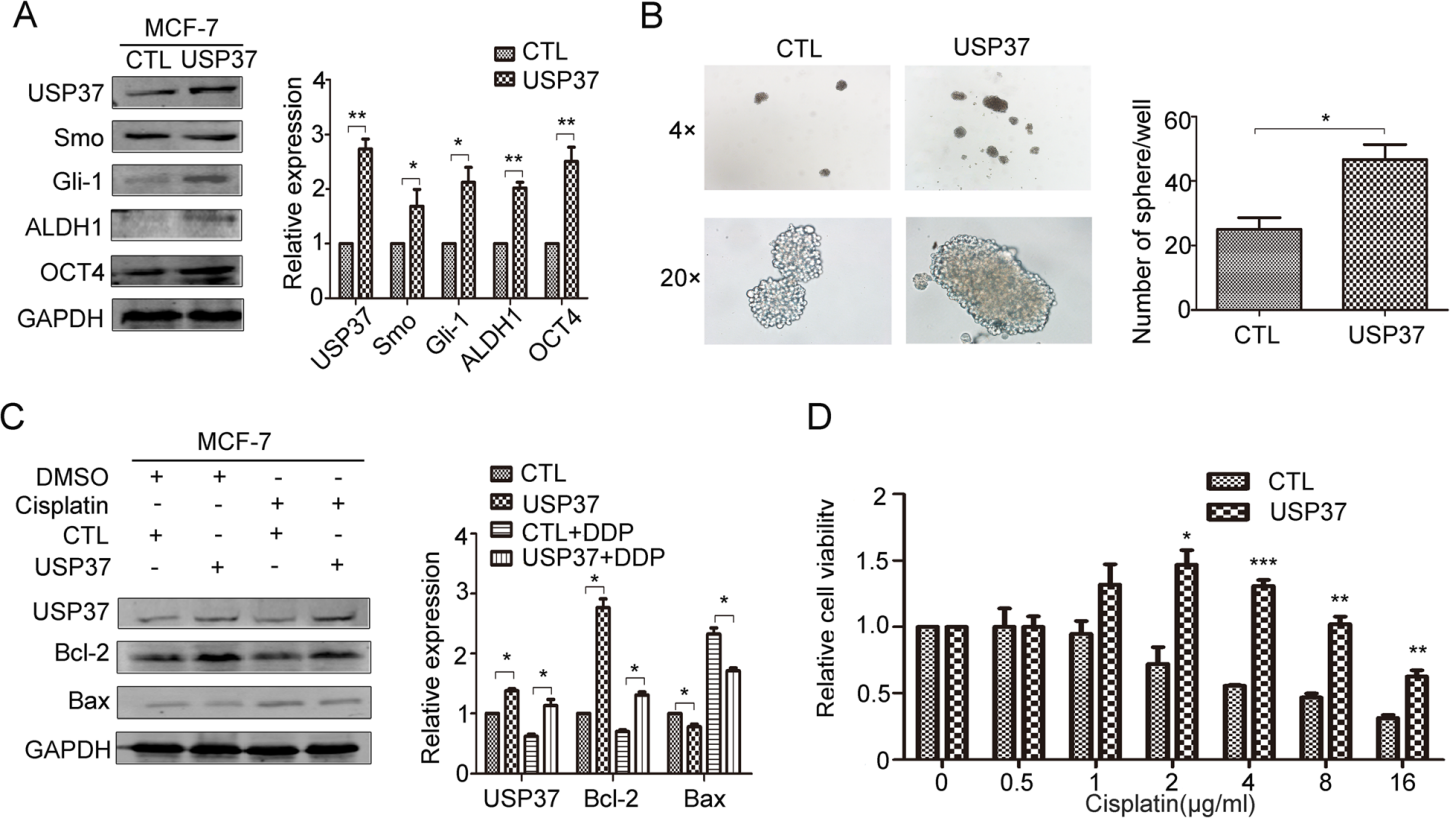
Overexpression USP37 elevates cell self-renewal and chemo-resistance to cisplatin. **a** Expressions of USP37, Smo, Gli-1, ALDH1 and OCT4 were detected in MCF-7-CTL or MCF-7-USP37 cells by western blotting. *P < 0.05, **P < 0.01. **b** Comparison of mammosphere formation among MCF-7-CTL or MCF-7-USP37 cells. *P < 0.05. **c** MCF-7-CTL and MCF-7-USP37 cells were treated with cisplatin (1 μg/ml) for 48 h, then treated cells were harvested in order to detect the levels of USP37, Bcl-2 and Bax. GAPDH was examined as a loading control. *P < 0.05. **d** CCK-8 assay showed that cell viability was enhanced in MCF-7 after USP37 upregulation compared to the control group. *P < 0.05, **P < 0.01, ***P < 0.01

Since USP37 was highly expressed in breast cancer stem cells, we further examined whether USP37 played a role in breast cancer stem cells behavior. We knocked down USP37 in MCF-7 and MDA-MB-231 cells via infection with lentivirus expressing USP37#2 shRNA or control lentivirus (shScramble); we confirmed USP37 gene expression by examining protein levels (Fig. 4a, b). The Hh pathway is considered to control the self-renewal of CSCs in breast cancer [25]; therefore, we tested the expression of representative Hh signaling factors (smoothened and Gli-1) and main stem cell markers (ALDH1 and OCT4) in USP37 knockdown cells and control cells by western blotting analysis. We observed that expression of smoothened, Gli-1, ALDH1 and OCT4 were decreased in USP37 knockdown cells compared with their counterpart cells, indicating that inhibited stemness was accompanied by a decrease in USP37 expression (Fig. 4a and b). In addition, USP37 knockdown significantly inhibited the formation of spheroids, as well as their size and volume, compared to the control cells in MCF-7 and MDA-MB-231 cells (Fig. 4c and d). With regard to chemical sensitivity, the cytotoxic effects of different concentrations of cisplatin on USP37 knockdown cells and their respective control cells were detected after 48 h treatment of cisplatin by CCK-8 assays. The results indicated that USP37 knockdown cells had increased sensitivity to cisplatin-induced growth inhibition and as evidenced by decreased colony formation compared with the control MCF-7 and MDA-MB-231 cells (Fig. 4f–i, k and l). Furthermore, the expression of Smo, Gli-1, ALDH1 and OCT4 were elevated in USP37 overexpression plasmid transfected MCF-7 cells compared with the control cells (Fig. 5a). USP37 overexpression also increased the formation of spheroid and chemo-resistance to cisplatin-induced growth inhibition (Fig. 5b, d).

To further explore the mechanism involved in cisplatin-induced apoptosis caused by USP37 knockdown, we detected apoptosis-related markers Bcl-2, Bax and cleaved caspase-9 in treated cells. We observed the upregulation of Bax and cleaved caspase-9 and downregulation of Bcl-2 in MCF-7 cells and MDA-MB-231 cells after transfection with USP37 shRNA and/ or treatment with cisplatin for 48 h (Fig. 4e, j). In the Bcl-2 protein family, the Bcl-2/Bax ratio has been shown to affect apoptosis induction [26, 27]. Our data indicated that USP37#2 shRNA combined with cisplatin induced the cell apoptosis with an underlying decrease in the Bcl-2/Bax ratio. Consistent with our hypothesis, an increase in the Bcl-2/Bax ratio might be involved in anti-apoptosis activity induced by USP37 upregulation treatment with cisplatin in MCF-7 cells (Fig. 5c). These data demonstrated that USP37 knockdown induced drug sensitivity by dislocating intracellular apoptosis-related proteins.

### Downregulation of USP37 inhibits stemness, cell invasion and EMT via hedgehog signaling pathway in breast cancer

**Fig. 6:**
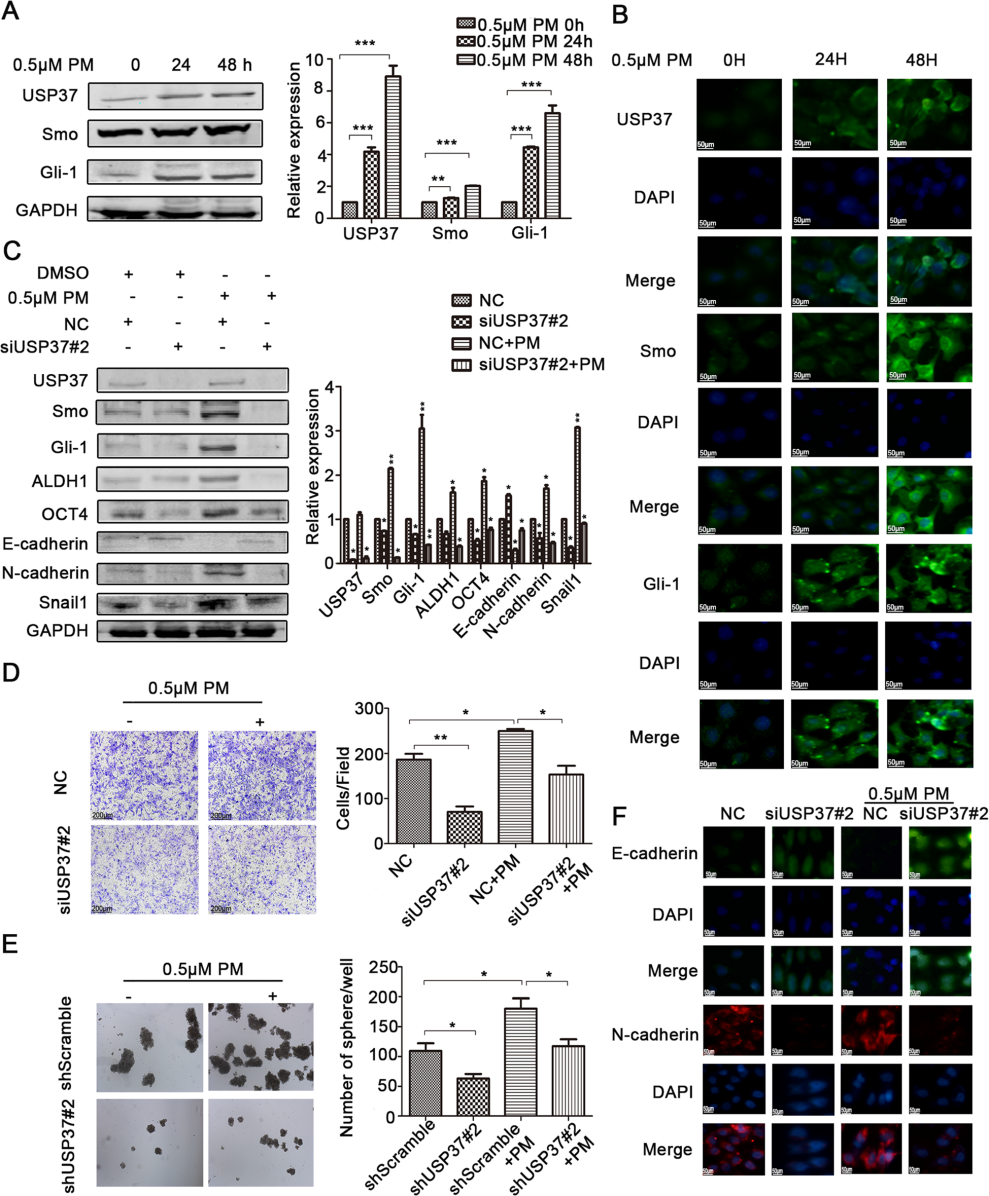
USP37 knockdown inhibits stemness, cell invasion and EMT via Hedgehog signaling pathway in breast cancer. **a**, **b** MCF-7 cells were incubated with 0.5 μM purmorphamine for 24 and 48 h. **a** Hedgehog pathway constituents were examined via western blotting. GAPDH was examined as a loading control. **P < 0.01,***P < 0.001. **b** Immunofluorescence staining images of MCF-7 cells showed the expression of USP37 and Hedgehog pathway constituents. **c** Protein levels of USP37, Smo, Gli-1, ALDH1, OCT4, E-cadherin, N-cadherin, Snail1 as detected by western blotting after the NC siRNA group or the USP37 siRNA#2 group was treated with 0.5 μM purmorphamine for 48 h. GAPDH was examined as a loading control. **P < 0.01,***P < 0.001. **d** Cell invasion capacity of the NC siRNA group or the USP37 siRNA#2 group treated with 0.5 μM purmorphamine (Scale bar: 200 μm). **e** Spheroid formation capacity of MCF-7-ShScramble or MCF-7-shUSP37#2 cells treated with 0.5 μM purmorphamine (original magnification, 4×). **f** Immunofluorescence staining of E-cadherin and N-cadherin after the NC siRNA group or the USP37 siRNA#2 group treated with 0.5 μM purmorphamine for 48 h. (Scale bar: 50 μm). *P < 0.05, **P < 0.01

It has been verified that tightly controlled Hh pathway ensures proper development and averts tumor formation in the mammary gland [10]. In ER-positive breast cancer, Hh pathway contributes to the maintenance and regulation of CSC invasion and EMT [28]. We were able to further determine the effect of USP37 on the stemness, cell invasion and EMT via Hh signaling pathway. Purmorphamine (PM) is considered to be an Hh agonist. Smo, Gli-1 and USP37 expression post-PM treatment was examined by western blotting analysis and immunofluorescence analysis. Interestingly, our data indicated that there was a time-dependent increase in the expression of Smo, Gli-1 and USP37 expression following 24 h, 48 h PM treatment (0.5 μM) (Fig. 6a). Immunofluorescence assays showed similar results (Fig. 6b).

These findings are further supported by the ability of USP37 to participate in Hh signaling pathway in breast cancer. Figure 6c shows that silencing of USP37 expression could reverse the positive effects of PM on the Hh pathway. Moreover, the effect of downregulating USP37 on CSC traits, including the formation of spheroid, BCSCs markers and cell invasion, was impaired after PM treatment (Fig. 6c, d and e and Additional file 2: Figure S1). Silencing of USP37 expression induced MET including upregulated E-cadherin expression and downregulated N-cadherin expression, while PM reversed MET in silenced USP37 cells. Similar results were observed with immunofluorescence assay (Fig. 6f). Collectively, these data indicate that downregulation of USP37 expression was involved in the attenuation of the BCSCs stemness, cell invasion and EMT by suppression of the Hh pathway.

### USP37 interacts with and stabilizes Gli-1 protein

**Fig. 7:**
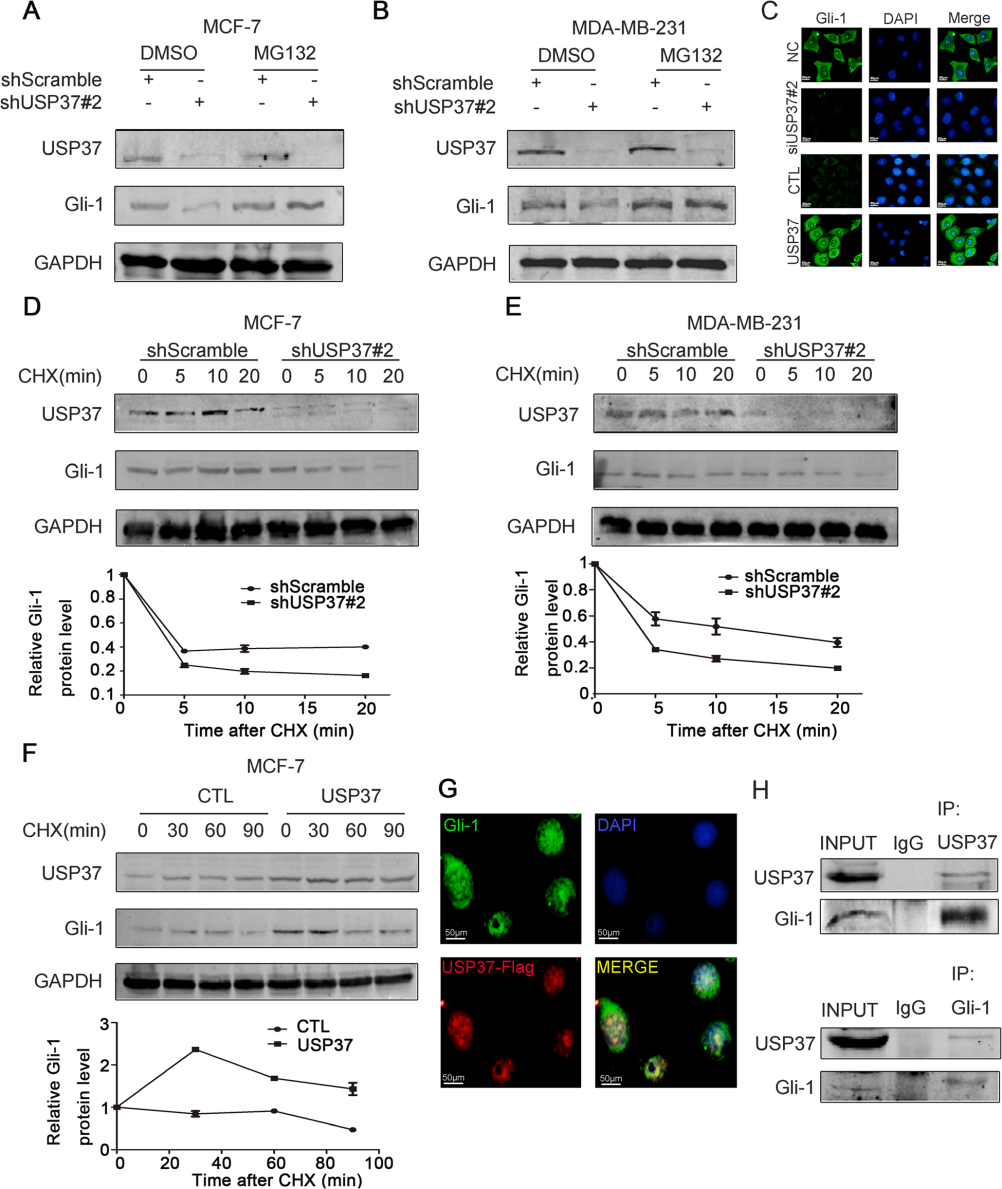
USP37 interacts with and stabilizes Gli-1 protein. **a**, **b** MCF-7 and MDA-MB-231 cells were transfected with shScramble and shUSP37#2, respectively, then treated with 10 μM of MG132 4 h before harvesting. **c** Immunofluorescence staining of Gli-1 after USP37 knockdown or USP37 overexpression (Scale bar: 50 μm). **d**, **e** Cells transfected with shScramble and shUSP37#2 were treated with 50 μg/ml of CHX and harvested at the indicated time point. **f** MCF-7-CTL and MCF-7-USP37 cells were incubated with 50 μg/ml of CHX and harvested at the indicated time point. Gli-1 expression was analyzed by western blotting and quantified with Image-Pro Plus 6.0 software. **g** Exogenous USP37 and Gli-1 magnified strong, positive overlapping expression in MCF-7 cells by immunofluorescent staining (Scale bar: 50 μm). **h** CO-IP assay showed the interaction between endogenous USP37 and Gli-1 in MCF-7 cells

Given that USP37 expression was significantly associated with the Hh pathway, we detected whether endogenous USP37 could also regulate Gli-1 stability. Endogenous USP37 was depleted by lentivirus expressing USP37#2 shRNA in MCF-7 and MDA-MB-231 cell lines. The effect of USP37 knockdown on Gli-1 was evidently reversed by MG132, which is a protease inhibitor (Fig. 7a and b). Furthermore, knockdown of USP37 expression significantly decreased the stability of Gli-1 protein both in MCF-7 and MDA-MB-231 cells (Fig. 7d and e). Additionally, the cycloheximide (CHX) chase assay was used to assess MCF-7 breast cancer cell line for the effect of USP37 upregulation on endogenous Gli-1 protein. As shown in Fig. 7f, upregulation of USP37 expression consistently stabilized Gli-1 protein. Immunofluorescence staining assays showed that USP37 could regulate Gli-1 expression (Fig. 7c). Co-IP assay indicated that USP37 interacted with Gli-1(Fig. 7h). We also found overlapping expression of exogenous USP37 and Gli-1 by immunofluorescence staining (Fig. 7g). Together, these data suggest endogenous USP37 may regulate Gli-1 protein stability in breast cancer.

### USP37 knockdown inhibits tumorigenicity and increases sensitivity to cisplatin in vivo

**Fig. 8:**
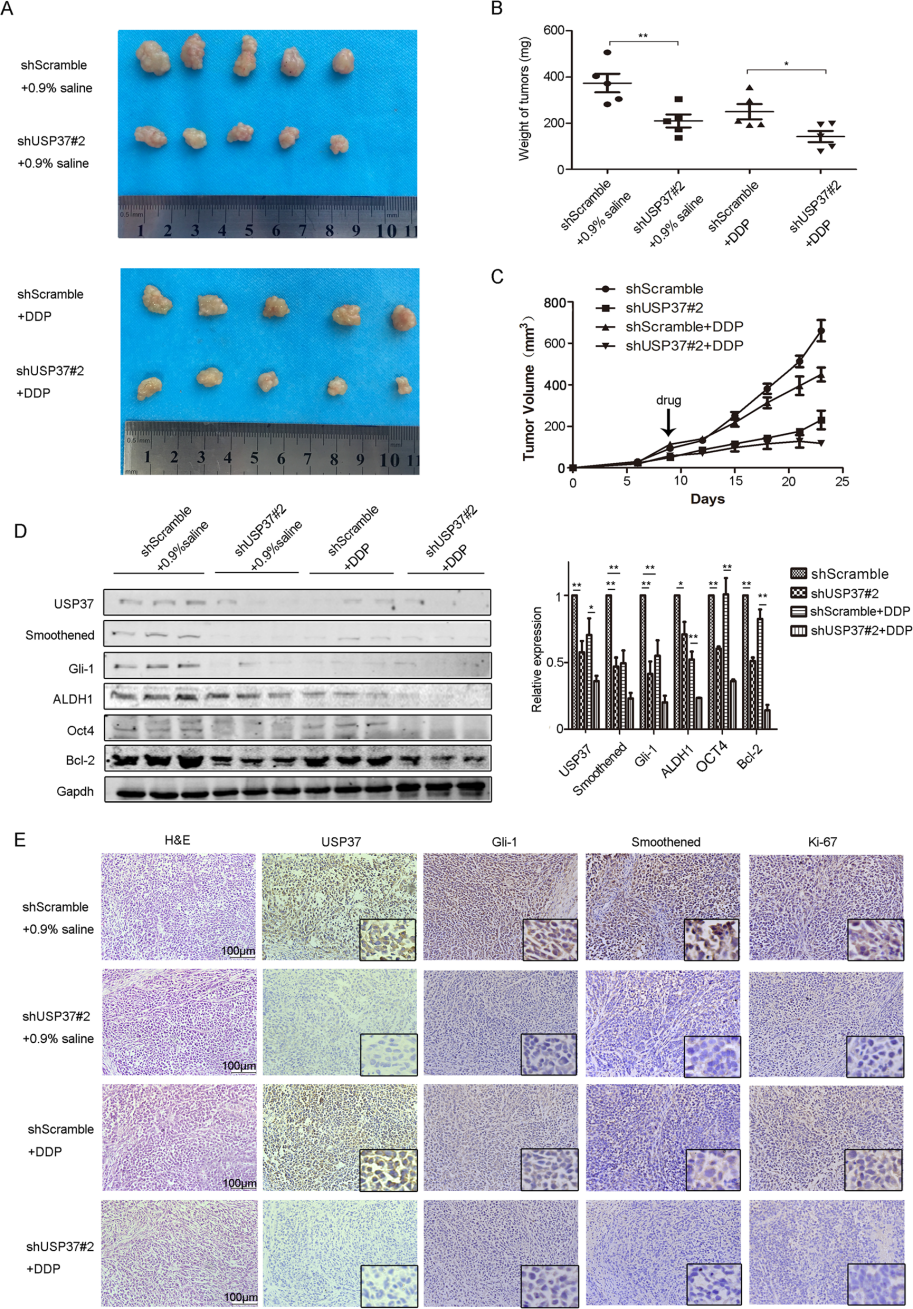
USP37 knockdown inhibits tumorigenicity and increases sensitivity to cisplatin in vivo. **a**, **b**, **c** MCF-7 cells transfected with shScramble or shUSP37#2 were subcutaneously injected into BALB/c nude mice treated with 0.9% saline or cisplatin. Tumors were excised from nude mice at day 28. **a** Images of tumor xenografts and **b** tumor weight for tumor xenografts treated with 0.9% saline or cisplatin are shown above. *P < 0.05, **P < 0.01. **c** Tumor growth curves were drawn every other day up to day 25. **d** Western blotting analysis of USP37, Smoothened, Gli-1, ALDH1, OCT4 and Bcl-2 in tumor xenografts. GAPDH was used as a loading control. *P < 0.05, **P < 0.01. **e** HE staining and immunohistochemical detection of USP37, Smoothened, Gli-1, Ki-67 in tumor xenografts. Scale bar: 100 μm

To further assess the anti-tumor effect of USP37 downregulation on tumor growth and cisplatin sensitivity in vivo, we established a xenograft tumor models by subcutaneously injecting breast cancer MCF-7 cells, which had been transfected with USP37#2 shRNA or scramble shRNA, into the mammary gland of nude mice (Fig. 8a). Tumor volume and weight measurements were analyzed. Compared with the scramble shRNA control group, the volume and weight of USP37 shRNA group increased at slower rate (Fig. 8b, c). The average tumor volume and weight of cisplatin-treated USP37#2 shRNA tumors was evidently smaller than that of cisplatin-treated tumors or USP37#2 shRNA tumors. We found that lower expression level of USP37 obviously impaired the expression of Hh targets (Smo and Gli-1) and cell proliferation marker Ki-67 in the tumor tissues as seen by immunohistochemical staining (Fig. 8e). Consistent with these results, tissues formed from cisplatin-treated USP37 knockdown cells showed the lowest expression levels of Smo, Gli-1, ALDH1, OCT-4 and Bcl-2 than other tissues by western blotting analysis (Fig. 8d). These results indicated that USP37 downregulation attenuates breast cancer progression and enhances sensitivity to cisplatin in vivo.

## Discussion

DUBs have been shown to participate in ubiquitin cleaving from ubiquitin conjugated protein substrates [29, 30]. USP37, a novel DUB, contains an insert between its catalytic lobe and its ubiquitin-binding lobe while its function could prevent 14-3-3γ degradation, which might contribute to malignant transformation by MAPK signaling [31, 32]. Subsequently, it was verified that USP37 expression modulated the oncogenic fusion protein PLZF/RARA stability and cell transformation potential in PLZF/RARA-associated acute promyelocytic leukemia [33]. Recently, clinicopathological analysis confirmed that USP37 was a poor prognostic factor in breast cancer [16]. However, there was no direct evidence to identify the carcinogenic mechanism of the USP37 gene in breast cancer. In this study, we have demonstrated overexpression of USP37 in Luminal B subtype breast cancer was a predictor of poor outcomes in breast cancer. Supporting our results, previous clinicopathological analysis has demonstrated that overexpressed USP37 is considered to be a poor prognosis in breast cancer [16]. Additionally, GSEA analysis on the TCGA dataset implicated that USP37 expression was positively associated with metastasis, cell growth and anti-apoptosis. Therefore, USP37 levels could potentially serve as a specific oncogene involved in breast carcinoma progression.

Cancer stem cells (CSCs) are endowed with stem cell properties, which include the ability to self-renew and differentiate by symmetrical or asymmetrical cell division. CSCs self-renewal of the cellular population and generation of progenitor cells have been shown to resist radiation and chemotherapy. Therefore, these cells are commonly deemed to be crucial target for cancer therapy [34, 35]. It was verified that CSCs could be maintained in an undifferentiated status and induce tumor-sphere formation in defined serum-free medium [36]. However, limited markers were utilized for the identification of breast cancer stem cells. While researchers have demonstrated that CD44 and ALDH1 are critical biomarkers to identify BCSCs from breast cancer populations [37, 38], our data suggested that the USP37 gene was significantly associated with CSC properties, such as self-renewal, treatment resistance and EMT phenotype as well. Interestingly, elevated mRNA expression of USP37 was detected in CD24^−^/CD44^+^ cells and ALDH1^+^ cells; the protein expression of USP37 was obviously elevated in spheres compared to adherent cells. Knockdown of USP37 suppressed mammospheres formation and inhibited cancer stem markers, such as ALDH1and OCT4 in breast cancer cells. It has been reported that USP37 participates in regulation of CSCs-related proteins, such as SOX2 and c-myc [20, 21]. Our data further indicated that knockdown of USP37 by constructed lentiviral system weakened the stemness in MCF-7 and MDA-MB-231 cells via inhibiting the expression levels of ALDH1 and OCT4. CSCs are considered to be “bad seeds” due to their drug resistance caused by imbalanced pathway and epigenetics in cancer [39]. As shown above, we detected that knockdown of USP37 in breast cancer cells could promote the sensitivity to cisplatin-induced cell death by the CCK-8 assay and colony formation assays. The Bax/Bcl-2 ratio is a commonly used method to determine whether intracellular apoptosis system is activated [40]. We detected that USP37#2 shRNA combined with cisplatin treatment induced cell apoptosis with an underlying decrease in Bcl-2/Bax ratio. On the contrary, the upregulation of USP37 reversed this phenomenon. USP37 knockdown suppressed stemness and chemo-resistance, which may assist clinical oncologists in designing and testing novel therapeutic strategies. In summary, we suggest that USP37 gene expression confers the stemness and potentially acts as a critical marker of CSCs in breast cancer.

The process of EMT was involved in the acquisition of aggressive cellular traits, including motility, invasiveness and anti-apoptosis, resulting in the dissemination of cells and colonization in distance tissues [6, 41, 42]. Here, we showed that USP37 siRNA treatment stimulated the expression of epithelial marker (E-cadherin) but decreased the expression of three known inducers of EMT (Snail1, N-cadherin and Vimentin) (Fig. 6a). Moreover, we found that knockdown of USP37 suppressed cell migration and invasion in breast cancer cells. On the contrary, upregulation of USP37 promoted EMT, migration and invasion. Accumulation evidence has indicated that tumor cells undergoing EMT process are endowed with the trait of cancer stem-like cells [43], which further speculated USP37 as a CSC marker of breast cancer. In summary, our data showed that USP37 could regulate the migration, invasion, EMT of breast cancer cells.

The Hh pathway was found to be required for the maintenance of breast CSCs traits, tumor formation and the EMT [10]. Normally, the ligand binding of Patched (Ptch1), a 12-pass transmembrane receptor of Sonic Hedgehog (SHH), can activate zinc finger transcription factor (Gli-1). It has been suggested that ectopic expression of Gli-1 upregulates expression of the transcription factor Snail1 accompanied with a decrease in E-cadherin, a characteristic of EMT [24]. In fact, Snail expression promotes EMT via repressing E-cadherin [44]. Hh signaling pathway modulates EMT indirectly via Snail [45]. Therefore, it is a promising research to investigate whether USP37 promoted the activation of the Hh pathway.

Our findings suggested that the post transcriptional levels of Smo and Gli-1 were decreased in vitro and in vivo. Upregulation of USP37 could enhance protein expression levels of Smo and Gli-1. In order to understand the mechanism of USP37-induced EMT and stemness via the Hh pathway, we treated MCF-7 cells with PM. We found that activation of Hh signaling pathway was accompanied by elevated expression of USP37 gene as visualized by western blotting and immunofluorescence assay. Furthermore, the effect USP37 downregulation on CSCs traits including the formation of spheroids, BCSCs markers and cell invasion were impaired after PM treatment. Meanwhile, knockdown of USP37 reversed the effect of PM on the EMT markers. These data confirm that USP37 mediates breast cancer stem-like properties, cell invasion and EMT via the Hh pathway. Gli-1 acts exclusively as a transcriptional activator in the Hh pathway and signal outcomes are determined by the balance of activated and inhibitive Gli proteins [46]. Remarkably, we found that USP37 could regulate and stabilize the protein level of Gli-1 (Additional file 2 : Figure S1). As a deubiquitinase, USP37 is involved in the regulation of multiple proteins by deubiquitination, including P27, Cdt1, PLZF/RARA and 14-3-3γ [31, 33, 47, 48]. Future work should aim to determine whether USP37 could stabilize Gli-1 through deubiquitination.

## Conclusions

In conclusion, our research demonstrated that USP37 was highly expressed in breast CSCs and was correlated with poor prognosis in breast cancer patients. Knockdown of USP37 expression hampered cell invasion, stemness, EMT and also resulted in the drug sensitivity to cisplatin. Mechanically, USP37 could orchestrate the stemness, cell invasion and EMT via activation of Hedgehog pathway. Further studies detected that USP37 also interacted with and stabilized Gli-1 protein, which is the main activator of Hedgehog target gene. These results indicate that USP37 is essential in the regulation of breast cancer progression suggesting an experimental basis for its use as a cancer biomarker and expands its potential clinical value.

## Additional files

Additional file 1: **Table S1.** Sequences of primer, siRNA and shRNA. (DOCX 14 kb)

Additional file 2: **Figure S1.** Protein level of OCT4 as detected by western blotting after the NC siRNA group or the USP37 siRNA#2 group was treated with 0.5 µM purmorphamine for 48 h. GAPDH was examined as a loading control. (DOCX 80 kb)

## Abbreviations

ALDH1: Aldehyde dehydrogenase1
BC: Breast cancer
BCSCs: Breast cancer stem cells
DUBs: Deubiquitinating enzymes
EMT: Epithelial-mesenchymal transition
Gli-1: Glioma-associated oncogene 1
GSEA: Gene set enrichment analysis
Hh: Hedgehog
MACS: Magnetic activated cell sorting
MET: Mesenchymal–epithelial transition
PM: Purmorphamine
Smo: Smoothened
USP37: Ubiquitin specific peptidase 37
