# Correction to: Abnormally elevated USP37 expression in breast cancer stem cells regulates stemness, epithelial-mesenchymal transition and cisplatin sensitivity

**DOI:** 10.1186/s13046-021-02158-4

**Published:** 2021-11-09

**Authors:** Tao Qin, Bai Li, Xiaoyue Feng, Shujun Fan, Lei Liu, Dandan Liu, Jun Mao, Ying Lu, Jinfeng Yang, Xiaotang Yu, Qingqing Zhang, Jun Zhang, Bo Song, Man Li, Lianhong Li

**Affiliations:** 1 Department of Pathology, Dalian Medical University, Dalian, 116044 People’s Republic of China; 2 The Key Laboratory of Tumor Stem Cell Research of Liaoning Province, Dalian Medical University, Dalian, 116044 People’s Republic of China; 3 Department of Urology, The First Affiliated Hospital of Dalian Medical University, Dalian, People’s Republic of China; 4 Teaching Laboratory of Morphology, Dalian Medical University, Dalian, 116044 People’s Republic of China; 5 Department of Pathology, Xiangyang Central Hospital, Xiangyang, 441000 People’s Republic of China; 6 Department of Dean, Dalian Medical University, Dalian, 116044 People’s Republic of China; 7 Department of Oncology, The Second Affiliated Hospital of Dalian Medical University, Dalian, 116023 Liaoning Province People’s Republic of China

**Correction to: J Exp Clin Cancer Res 37, 287 (2018)**

**https://doi.org/10.1186/s13046-018-0934-9**

Following publication of the original article [1], the authors identified minor errors in Fig. 6; specifically, in Fig. 6D, the incorrect transwell analysis image was used for the siUSP37#2 group without purmorphamine (bottom left image).

**Fig. 6:**
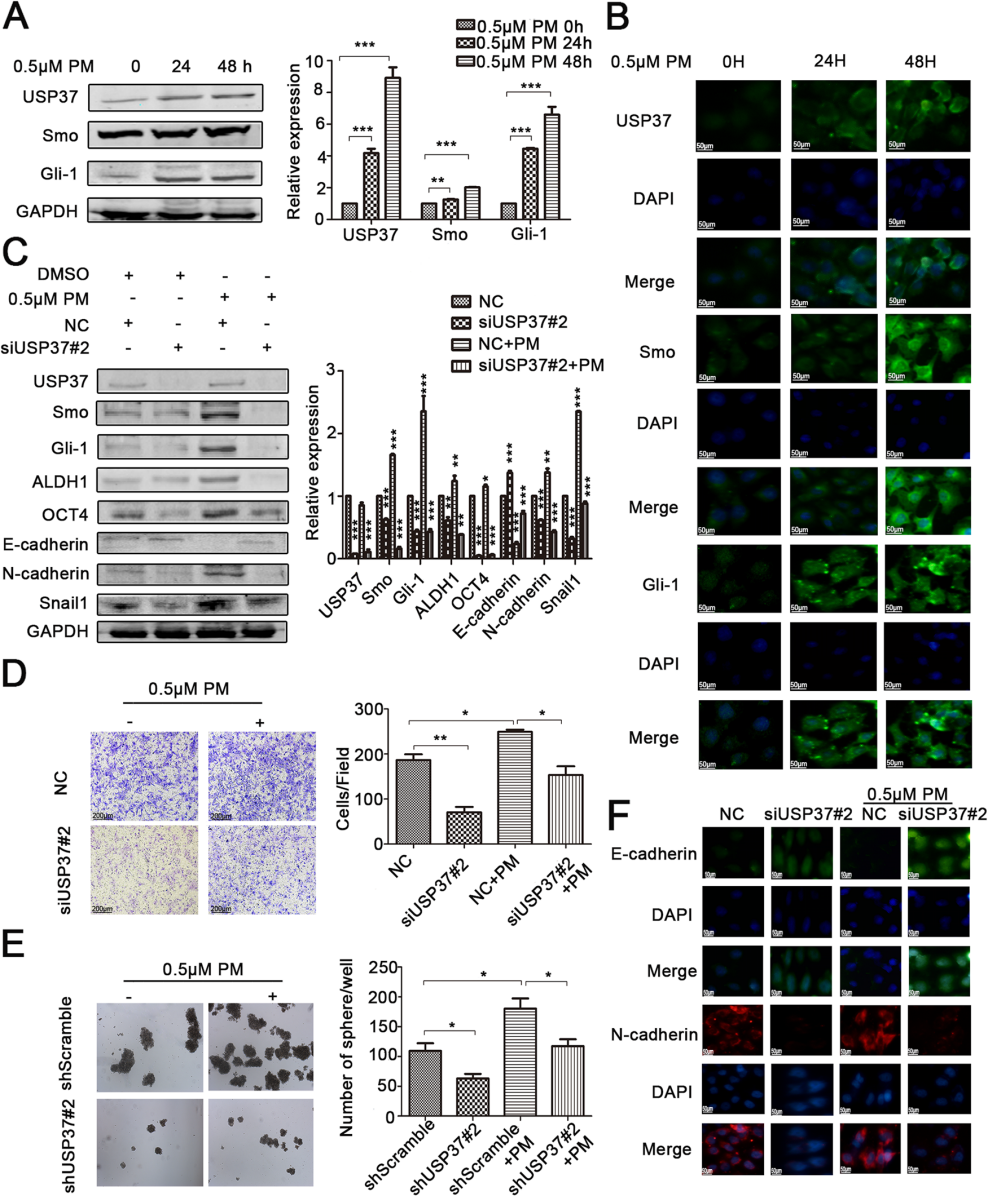
USP37 knockdown inhibits stemness, cell invasion and EMT via Hedgehog signaling pathway in breast cancer. **a**, **b** MCF-7 cells were incubated with 0.5 μM purmorphamine for 24 and 48 h. **a** Hedgehog pathway constituents were examined via western blotting. GAPDH was examined as a loading control. **P < 0.01,***P < 0.001. **b** Immunofluorescence staining images of MCF-7 cells showed the expression of USP37 and Hedgehog pathway constituents. **c** Protein levels of USP37, Smo, Gli-1, ALDH1, OCT4, E-cadherin, N-cadherin, Snail1 as detected by western blotting after the NC siRNA group or the USP37 siRNA#2 group was treated with 0.5 μM purmorphamine for 48 h. GAPDH was examined as a loading control. **P < 0.01,***P < 0.001. **d** Cell invasion capacity of the NC siRNA group or the USP37 siRNA#2 group treated with 0.5 μM purmorphamine (Scale bar: 200 μm). **e** Spheroid formation capacity of MCF-7-ShScramble or MCF-7-shUSP37#2 cells treated with 0.5 μM purmorphamine (original magnification, 4×). **f** Immunofluorescence staining of E-cadherin and N-cadherin after the NC siRNA group or the USP37 siRNA#2 group treated with 0.5 μM purmorphamine for 48 h. (Scale bar: 50 μm). *P < 0.05, **P < 0.01

The corrected figure is provided here. The correction does not have any effect on the results or conclusions of the paper. The original article has been corrected.
