# Supplementary material for: Abnormally elevated USP37 expression in breast cancer stem cells regulates stemness, epithelial-mesenchymal transition and cisplatin sensitivity
Source: J Exp Clin Cancer Res. 2018 Nov 27;37:287. doi: 10.1186/s13046-018-0934-9 (PMC6258492; doi:10.1186/s13046-018-0934-9)
Supplement: Supplementary file 1 — Table S1. Sequences of primer, siRNA and shRNA. (DOCX 14 kb) [file 13046_2018_934_MOESM1_ESM.docx]

**Table S1: Sequences of primer, siRNA and shRNA.**

| **Gene** | **Sequences** |
| --- | --- |
| USP37 | F, 5’-GGC AGC AAG TCA TCA TTC CA-3’  R, 5’-GGC TGG TGA TGC AGG AAT TC-3’ |
| ALDH1 | F,5’-TGCAGGTTGGGCTGACAA-3’  R,5’-GCAGGCCCTATCTTCCAAATG-3’ |
| CD44 | F,5’-CTGAGCCTGGCGCAGATC-3’  R,5’-CTCCATCTGGGCCATTGT-3’ |
| CD24 | F,5’-GAACTTCAAGTAACTCCTCCCAGAGT-3’  R,5’-AGAGAGAGTGAGACCACGAAGGAGACT-3’ |
| pEZ-M35 USP37 plasmid | 5’-AGGCACTGGGCAGGTAAG-3’  5’-GTGGCACCTTCCAGGGTC-3’ |
| *siUSP37#1* | 5’-GGA GGA ATT CCA AGG ATAT-3’ |
| *siUSP37#2* | 5’-CCG AAG AAC TGG AGT ATTC-3’ |
| *siUSP37#3* | 5’-CCT AGT AGT TCA CTA CAAT-3’ |
| *shUSP37#2* | 5’-CCG AAG AAC TGG AGT ATTC-3’ |
