# Supplementary figures and images for: Abnormally elevated USP37 expression in breast cancer stem cells regulates stemness, epithelial-mesenchymal transition and cisplatin sensitivity
Source: J Exp Clin Cancer Res. 2018 Nov 27;37:287. doi: 10.1186/s13046-018-0934-9 (PMC6258492; doi:10.1186/s13046-018-0934-9)

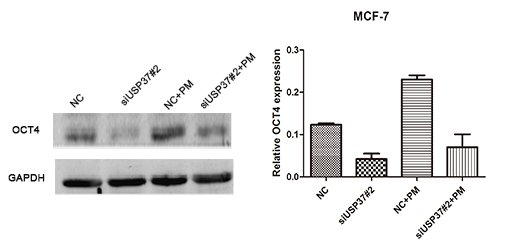

Supplement: Supplementary file 2 — Figure S1. Protein level of OCT4 as detected by western blotting after the NC siRNA group or the USP37 siRNA#2 group was treated with 0.5 µM purmorphamine for 48 h. GAPDH was examined as a loading control. (DOCX 80 kb) [file 13046_2018_934_MOESM2_ESM.docx]
